# Distinct neonatal hyperammonemia and liver synthesis dysfunction: case report of a severe MEGDHEL syndrome

**DOI:** 10.3389/fped.2024.1278047

**Published:** 2024-02-20

**Authors:** Ina Kirchberg, Elke Lainka, Andrea Gangfuß, Alma Kuechler, Fabian Baertling, Lea D. Schlieben, Dominic Lenz, Eva Tschiedel

**Affiliations:** ^1^ Department of Pediatric Intensive Care, Children’s Hospital, University of Duisburg-Essen, Essen, Germany; ^2^ Department of Pediatric Gastroenterology, Hepatology, and Transplant Medicine, Children’s Hospital, University Duisburg-Essen, Essen, Germany; ^3^ Department of Paediatric Neurology, Centre for Neuromuscular Disorders, Centre for Translational Neuro- and Behavioural Sciences, University Duisburg-Essen, Essen, Germany; ^4^ Institute of Human Genetics, University Hospital Essen, Essen, Germany; ^5^ Department of General Pediatrics, Neonatology and Pediatric Cardiology, University Children’s Hospital Duesseldorf, Heinrich Heine University, Dusseldorf, Germany; ^6^ School of Medicine, Institute of Human Genetics, Klinikum rechts der Isar, Technical University of Munich, Munich, Germany; ^7^ Institute of Neurogenomics, Computational Health Centre, Helmholtz Zentrum Muenchen, Neuherberg, Germany; ^8^ Division of Pediatric Neurology and Metabolic Medicine, Centre for Child and Adolescent Medicine, Heidelberg University, Medical Faculty, University Hospital Heidelberg, Heidelberg, Germany

**Keywords:** MEGDHEL syndrome, SERAC1, hyperammonemia, liver failure, neurometabolic disorder, lactic acidosis

## Abstract

**Background/purpose:**

MEGDHEL syndrome is a rare autosomal recessive metabolic disorder, which is characterized by 3-methylglutaconic aciduria with deafness-dystonia, hepatopathy, encephalopathy and Leigh-like syndrome. It is caused by biallelic pathogenic variants in the *SERAC1* gene. Due to the unspecific symptoms and the diverse manifestations of the clinical phenotype, the diagnosis is challenging. Infantile MEGDHEL syndrome often has a severe disease course with acute liver failure. Differentiation from other metabolic disorders is difficult and requires a multidisciplinary approach.

**Case presentation:**

A two-day-old small for gestational age neonate was admitted to our pediatric intensive care unit (PICU) due to severe liver failure with distinct hyperammonemia and hypoglycemia without elevation of transaminases or cholestasis. Due to high ammonia level, continuous hemodialysis was established immediately after admission. In addition, protein intake was stopped, and the patient anabolized with intravenous glucose. Temporary stabilization could be achieved after four days. In the further course, severe neurological and cardiocirculatory complications occurred, which ultimately led to the infant's death. In the metabolic diagnostics, a pronounced lactate acidosis and in urine an increased excretion of 3-methylglutaconic acid as well as other metabolites of mitochondrial energy metabolism has been the leading findings besides the hyperammonemia. Post-mortem trio whole genome analysis detected a homozygous pathogenic variant in *SERAC1* with evidence of SERAC1 deficiency leading to the diagnosis of infantile MEGDHEL syndrome.

**Conclusion:**

When pediatricians are faced with hepatopathy or even acute liver failure without elevation of transaminases or cholestasis in newborns, SERAC1 deficiency should be considered as a potential differential diagnosis. The initial treatment is based on the recommended management of suspected metabolic disorders. Even while no cure is available yet, patients should be offered proper supportive management through a multidisciplinary team. In addition, genetic confirmation of the diagnosis is important for the families, especially regarding further family planning.

If a newborn presents with hyperammonemia, hypoglycemia and impaired liver synthesis function without elevation of transaminases or cholestasis, the possible presence of MEGDHEL syndrome due to a SERAC1 mutation should be considered.

## Background

MEGDHEL (OMIM #614739) syndrome is a rare autosomal recessive metabolic disorder, which is characterized by 3-methylglutaconic aciduria with deafness-dystonia, hepatopathy, encephalopathy and Leigh-like syndrome. First description of 4 patients with MEGDEL (3-methylglutaconic aciduria with deafness-dystonia, encephalopathy and Leigh-like syndrome) was published by Wortmann et al. in (1). In the following years hepatopathy was incorporated into the acronym due to the observation of liver involvement as another clinical feature (2). MEGDHEL syndrome is caused by mutations in the *SERAC1* gene (OMIM *614725). SERAC1 deficiency can lead to different clinical phenotypes, varying according to the age of manifestation (3, 4). Most neonates develop problems during their first days of life. Symptoms are typically hypoglycemia, hyperammonemia, elevated serum lactate and sepsis-like picture not linked to infection (5).

We report a case of infantile-onset MEGDHEL syndrome due to a homozygous splice variant in *SERAC1* who was admitted to our pediatric intensive care unit (PICU) on the second day of life.

## Case report

A two-days-old small for gestational age (birth weight 2,580 g (5th percentile) neonate was transferred to our PICU for hemodialysis due to significant hyperammonemia (ammonia level 764 µg/dl). The patient was born as the first child (primigravida) of healthy, non-consanguineous parents. Postnatal cardiorespiratory adaptation was without complications; APGAR was 9/10/10 accordingly. The patient received a 2 mg oral dose vitamin K at birth. No congenital anomalies or dysmorphisms were noted. The maternity hospital is an urban hospital with level II perinatal care. The patient was initially breastfed and presented on the second day of life with recurrent hypothermia, poor sucking, and surpassing weight loss. Biochemical findings were partially compensated lactate acidosis (lactate 7.0 mmol/L, pH 7.14, PaCO_2_ 14.1 mmHg, BE −24.2 mmol/L) and hyperammonemia (589 µg/dl), with transaminases within the reference range.

Due to the distinct hyperammonemia the patient was transferred to a specific metabolic center requiring an ambulance ride of about 45 min. Initial management of hyperammonemia included the stop of protein intake, anabolization with glucose, support of nitrogen detoxification (sodium phenylacetate and sodium benzoate) and supplementation of carglum acid, L-arginine, L-carnitine, vitamin B12 and biotin. Despite intensive therapy, hyperammonemia increased to ammonia level 850 µg/dl and there were signs of acute liver failure (INR 2.1), which was not correctable by supplementation of vitamin K. Metabolic laboratory for investigation of inborn errors of metabolism including acylcarnitine profile analysis in dried blood spots, testing of organic acids in urine and analysis of plasma amino acids was performed and the patient was transferred to our PICU for hemodialysis.

**Figure 1:**
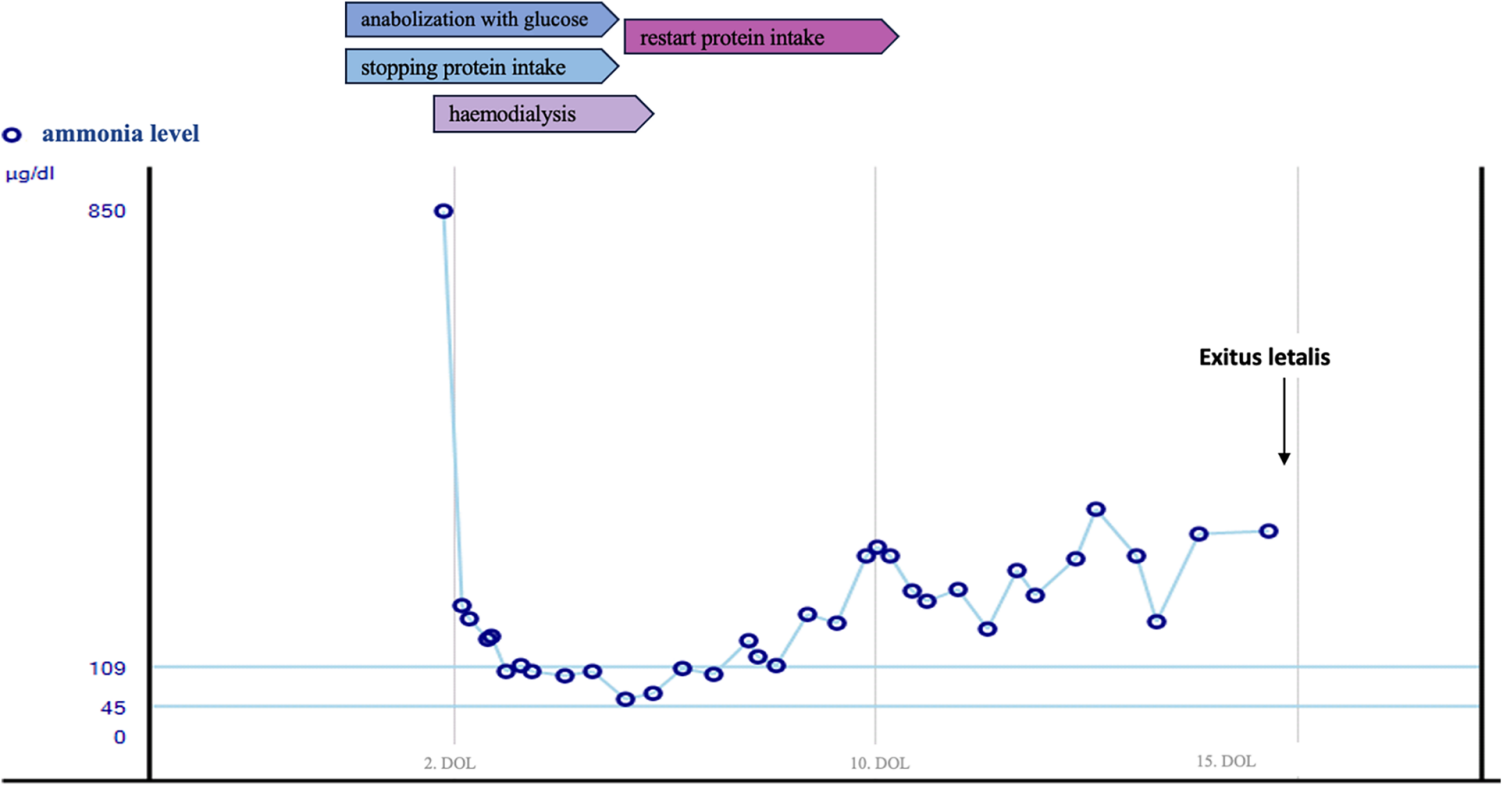
Course of serum ammonia level.

Examination findings: On admission to our PICU, the neonate presented in a severely reduced general condition, marked tachydyspnoea and reduced skin turgor. Other pediatric examination findings were unremarkable. Blood tests revealed evidence of acute liver failure: conjugated bilirubin 0.92 mg/dl (<0.2), total bilirubin 7.0 mg/dl (0.2–1), INR 2.45, aPTT 86.5 s (31–54), thrombin time 26.2 s (16.2–19.1), ammonia level 850 µg/dl (45–109) (Figure 1), while transaminases were still within the reference range [AST 86 U/L (<90), ALT 25 U/L (<60)].

After admission to our PICU intubation and emergency hemodialysis was performed via a Shaldon catheter. Within 7 h after admission the ammonia level reached normal ranges. Repeated supplementation with coagulation factors and fresh frozen plasma were necessary. During hemodialysis, the patient was catecholamine-dependent, therefore requiring noradrenaline max. 0.7 µg/kg/min iv and dobutamine max. 20 µg/kg/min iv. The initial abdominal ultrasound was suspicious for portal vein thrombosis. Etiologically, we assume that this is a complication of umbilical vein/arterial catheter. Partial recanalization was achieved after three days of heparin administration.

Protein fasting and anabolization by increased glucose intake (15 g/kg/d) and hydration were continued. In addition, the patient received L-arginine (2 mmol/kg/d iv), nitrogen scavengers (sodium benzoate (250 mg/kg/d iv), and carglumic acid (77 mg/kg/d PO) because a urea cycle disorder was suspected.

Biochemical results were neither suggestive for urea cycle disorder (standard values for plasma citrulline and arginine) nor for mitochondrial disease (standard values for plasma alanine and plasma proline). Even though also the investigation for mitochondrial fatty acid oxidation disorders by acylcarnitine profile proved unremarkable, L-carnitine (100 mg/kg/d iv) was administered until urine organic acids entirely excluded the prevalence of organic aciduria. Urinary organic acid analysis showed a massively increased excretion of lactate (20.108 mmol/mol creatinine; <413) and pyruvate (989 mmol/mol creatinine; <118), a significant ketonuria [3-hydroxybutyrate (1,042 mmol/mol creatinine; <385) and acetoacetate (3,348 mmol/mol creatinine; <150)] as well as secondary changes due to mitochondrial dysfunction. Among those, a significant increase of 3-methylglutaconic acid (3-MGA) (128 mmol/mol creatinine; <15) was noted. With a concomitant urine creatinine of 18.1 mg/dl, this was considered a significant 3-MGA aciduria (6). From this point on the main diagnostic hypothesis resulted being a mitochondrial metabolic disorder with 3-methylglutaconic aciduria and the patient was treated with riboflavin, thiamine and coenzyme Q 10 additively.

In the following days the patient's condition stabilized with sufficient spontaneous breathing. Hemodialysis was discontinued after 4 days.

Ammonia levels remained slightly elevated (150–290 µg/dl) (Figure 1). But re-introduction of protein intake, did not result in a further increase.

On the 13th day of life, a second deterioration occurred unexpectedly. A trigger could not be identified. Focal seizures occurred and anticonvulsive therapy was started. Cranial magnetic resonance imaging (MRI) showed multiple supratentorial medullary lesions, some with impaired diffusion. Due to the patient's critical condition, the standard hearing test was postponed. Reintubation was necessary, and liver synthesis as well as cardiovascular function decompensated again. Stabilization could not be achieved, and the patient dies at the age of 15 days.

Postmortem, trio-whole-genome sequencing was performed and confirmed the homozygous prevalence of a pathogenic splice variant in *SERAC1* (NM_032861.4: c.1888_1828 + 10delinsACCAACAGG).

## Discussion

MEGDHEL syndrome is a rare neurometabolic disorder. By 2017, 67 cases had been reported worldwide (2). The prevalence is estimated at 0.09:100,000 (7). MEGDHEL syndrome is caused by biallelic pathogenic variants in the *SERAC1* gene. It encodes Serine active site containing protein 1 (SERAC1), a phosphatidylglycerol remodeling protein (4). Deficiency leads to dysfunction of oxidative phosphorylation and intracellular cholesterol trafficking (8). Most common metabolic changes detected are neonatal hypoglycemia and severe neonatal liver dysfunction (about 50% of all cases), lactic acidosis (85%) and in nearly all patients a 3-MGA aciduria (2). Wortman et al. divided the clinical course of SERAC1 deficiency in three groups depending on the age of manifestation (3).

1. **Infantile, Severe MEGDHEL Syndrome**: Typical first symptoms are hypotonia and drinking failure, hypoglycemia, lactic acidosis as well as liver dysfunction or a sepsis-like clinical picture without infection (5). Liver failure can present as undulating increase of transaminases, prolonged hyperbilirubinemia up to severe liver failure (3). Then failure to thrive, various liver problems, progressive spasticity and sometimes epilepsy can follow. Survival varies enormously. Some children do not even survive the neonatal period. In the neonatal period, some succumb to multiorgan failure. In infancy, liver failure is a frequent cause of death whereas later in life some succumb to infections, especially pulmonary infections (3, 4).
2. **Milder Juvenile-Onset Complicated Hereditary Spastic Paraplegia (cHSP)**: Roeben et al. (9) present a family in which five of the six siblings had spasticity as well as non-progressive mild cognitive deficits.
3. **Adult-Onset Generalized Dystonia**: At present, there is only one case reported with mild psychomotor developmental deficits and generalized dystonia.

In the present case, we report on a patient with neonatal onset of MEGDHEL syndrome presenting at the 2nd day of life. However, due to the low birth weight of our patient it can be assumed that the initial onset had been intrauterine. Fellman et al. and Maas et al. also report two cases of infantile MEGDHEL Syndrome who were small for gestational age (2, 10). Pediatric acute liver failure (PALF) is a progressive, potentially lethal clinical condition mainly occurring in the first year of life and according to the PALF Study group defined as a rapidly progressive, potentially fatal clinical syndrome with (a) biochemical evidence of hepatocellular injury and (b) a coagulopathy that cannot be corrected with vitamin K alone with an INR ≥ 1.5 with hepatic encephalopathy or an INR ≥ 2.0 regardless of the presence of hepatic encephalopathy (11, 12). In neonates, the main causes are viral infections (e.g., HSV, Enterovirus), immunological [e.g., gestational alloimmune liver disease (GALD)], inherited metabolic diseases (e.g., urea cycle disorder) and hematological diseases (e.g., hemophagocytic lymphohistiocytosis). However, in about 50% of the cases the etiology remains unexplained (11, 13, 14).

On top of liver synthesis failure hyperammonemia was the most pressing problem of our patient and required emergency dialysis. Molla et al. also describe two neonates with MEGDHEL syndrome required dialysis treatment due to hyperammonemia. Both patients survived the neonatal period and are currently in follow up controls (15). In our case there was an immediate initiation of supportive therapy, diet modification and fast establishment of continuous veno-venous hemodiafiltration (CVVHDF). These interventions improved the patient's state of health temporarily. Nevertheless, our patient died unexpectedly from neurological and cardiorespiratory deterioration only a few days after initial stabilization. This underlines the unpredictability of this disease's course.

Portal vein thrombosis is not part of or explained by the diagnosis of MEGHDEL syndrome and must be assumed to be secondary complication probably triggered by one of the inserted central venous catheters.

Impaired liver function only seems to play a decisive role during the neonatal period. Although transiently elevated transaminases or functional limitations are still found at older ages, usually they are not life-threatening (2). As children get older, other problems come to the fore and multidisciplinary therapy is needed to treat spasticity, dysphagia, deafness, and seizures which may occur.

There is no curative treatment available yet. The therapy is exclusively supportive and depends on the symptoms of the patient. In previous reports, the initial use of protein restriction, supplementation of coenzyme Q_10_, biotin and riboflavin has been described (2, 15). It is recommended to carry out the stepwise emergency therapy in neonates suspected of having a metabolic disease. In case of severe neonatal hyperammonemia coordinated treatment should be administered immediately with the aim of rapidly lowering the ammonia levels (16).

With the onset of the first symptoms, it is essential to determine the cause of disease. Differential diagnosis of neonatal liver failure with hyperammonemia must include metabolic disorders, viral infections, neonatal hemochromatosis, and hematological causes like hemophagocytic lymphohistiocytosis (HL) (2, 4, 17).

In our case, an infection could be excluded. Also, the prevalence of GALD was very unlikely as this was the mother's first pregnancy. Laboratory findings gave no clue of HL. Therefore, we focused on congenital metabolic disorders. Hence, special metabolic diagnostics including determination of the acylcarnitine profile in a dried blot spot, plasma amino acids, and organic acids as well as orotic acid in urine should be carried out. If possible, samples should be collected before the transfusion of blood products.

The cerebral MRI of our patient showed non-specific medullary lesions matching with a metabolic disorder. However, these aren't pathognomonic and cannot be clearly assigned to the stages postulated by Wortmann et al. They report five stages of characteristic MRI patterns over the course of the disease (18). Like in Leigh syndrome bilateral lesions, particularly in the basal ganglia, can be seen. While the imaging in the neonatal period is usually still unremarkable, the first changes occur during the first year of life.

If clinical and metabolic findings suggest SERAC1 deficiency, molecular genetic testing needs to be initiated to establish the diagnosis. Single gene testing via Sanger sequencing is only indicated in case of familial known pathological variants. For all other patients, trio genome or exome sequencing is the gold standard. Genome or trio genome sequencing are options on a research basis. Advances in molecular genetic testing over the last decade have enabled rapid diagnosis in rare diseases and thus improved patient care and reduced the recurrence risk.

## Conclusion

When pediatricians are faced with hepatopathy with distinct hyperammonemia, hypoglycemia and impaired liver synthesis function without elevation of transaminases or cholestasis in newborns, SERAC1 deficiency should be considered as a differential diagnosis. Especially if organic acids in urine show an elevated excretion of 3-MGA, one should think about MEGDHEL syndrome as a possible cause.

## Data Availability

The original contributions presented in the study are included in the article/Supplementary Material, further inquiries can be directed to the corresponding author.
